# Sprouty4 at the crossroads of Trk neurotrophin receptor signaling suppression by glucocorticoids

**DOI:** 10.3389/fnmol.2023.1090824

**Published:** 2023-02-02

**Authors:** Facundo Ferrero Restelli, Fernando Federicci, Fernanda Ledda, Gustavo Paratcha

**Affiliations:** ^1^Division de Neurociencia Molecular y Celular, Instituto de Biología Celular y Neurociencias Prof. E. De Robertis (IBCN), CONICET, Universidad de Buenos Aires, Buenos Aires, Argentina; ^2^Fundación Instituto Leloir, Instituto de Investigaciones Bioquímicas de Buenos Aires, CONICET, Buenos Aires, Argentina

**Keywords:** neurotrophins, Trk receptors, glucocorticoids, Sprouty4, Erk/MAPK pathway, neuronal differentiation

## Abstract

Glucocorticoids (GC) affect neuronal plasticity, development and function of the nervous system by inhibiting neurotrophin-induced Trk signaling. It has been established that pretreatment with dexamethasone (DEX) restricts Neurotrophin-induced neurite outgrowth by inhibiting Trk-dependent activation of Ras-Erk1/2 signaling pathways. However, the precise molecular mechanism through which DEX interferes with neurotrophin signaling and Trk-mediated neurite outgrowth has not been clearly defined yet. Here, we observed that in PC12 cells DEX treatment promotes the transcription of Sprouty4, a regulatory molecule that is part of a negative feedback module that specifically abrogates Ras to Erk1/2 signaling in response to NGF. In line with this, either knockdown of *Sprouty4* or overexpression of a dominant negative form of Sprouty4 (Y53A), rescue the inhibition of NGF/TrkA-promoted neurite outgrowth and Erk1/2 phosphorylation induced by DEX. Likewise, treatment of hippocampal neurons with DEX induces the expression of Sprouty4 and its knockdown abrogates the inhibitory effect of DEX on primary neurite formation, dendrite branching and Erk1/2 activation induced by BDNF. Thus, these results suggest that the induction of *Sprouty4* mRNA by DEX translates into a significant inhibition of Trk to Erk1/2 signaling pathway. Together, these findings bring new insights into the crosstalk between DEX and neurotrophin signaling and demonstrate that Sprouty4 mediates the inhibitory effects of DEX on neurotrophin function.

## Introduction

Neurotrophin binding to receptor tyrosine kinases (RTKs) of the Trk family, and glucocorticoids (GC) acting through nuclear receptors, are ligand-receptor systems involved in the control of structural and functional plasticity of the nervous system (Liston and Gan, 2011; Suri and Vaidya, 2013). GC are steroid hormones that function as stress-response hormones that regulate gene expression in order to maintain critical homeostatic functions and acutely facilitate stress coping (Kadmiel and Cidlowski, 2013). However, chronic GC exposure has adverse impact on brain development, affecting neuronal connectivity in diverse brain regions and increasing the susceptibility to develop cognitive and mood disorders (Harris and Seckl, 2011; Joels, 2011; Liston and Gan, 2011; Sousa and Almeida, 2012; Vyas et al., 2016).

The neurotrophins constitute a structurally-related family of proteins represented by Nerve Growth Factor (NGF), Brain-Derived Neurotrophic Factor (BDNF), Neurotrophin-3 (NT3) and Neurotrophin-4 (NT4), which support the differentiation and survival of specific populations of sensory, sympathetic, and central nervous system neurons *via* the activation of their cell-surface receptor tyrosine kinase, TrkA, TrkB, and TrkC (Reichardt, 2006; De Vincenti et al., 2019). Notably, disruption of neurotrophin signaling in specific regions of the nervous system has been linked to the development of neurodegenerative disorders and hippocampal-dependent memory deficits (Baquet et al., 2004; Alsina et al., 2012b). Once activated, Trk receptors trigger intracellular signal transduction cascades, including those mediated by Ras/Mitogen-Activated Protein Kinase (MAPK), PI3-kinase (PI3K)/Akt, PLC and other pathways regulated by Rho family of small GTPases. At the same time, Trk receptors also trigger a complex chain of molecular events, named negative signaling, that reduce the strength and duration of positive signals to finally modulate neuronal physiology and plasticity (Alsina et al., 2012b).

Clinically, synthetic GC such as dexamethasone (DEX) are used for the suppression of immune rejection after organ transplantation and for the limitation of the spreading of neuronal damage that occurs after nerve injury. However, post-injury inflammatory control by DEX appears to be in conflict with neurotrophic factor-induced regenerative responses. Several lines of evidence reported opposite effects of GC and neurotrophins regarding to neuronal plasticity and axonal growth. Intriguingly, while DEX appears to promote regeneration and functional recovery of axons after nerve crush (Feng et al., 2014; Gao et al., 2017), GC have been reported to prevent BDNF-induced dendritic growth and synaptic maturation and function of developing hippocampal and cortical neurons by reducing MAPK signaling pathway (Kumamaru et al., 2008; Numakawa et al., 2009; Kawashima et al., 2010). Moreover, in an attempt to decipher the mechanism through which GC could promote depression and affect neurodevelopment, Terada and colleagues also demonstrated that DEX binding to glucocorticoid receptors (GR) restricts NGF-promoted neurite outgrowth through a mechanism that prevents PI3K-Akt and Ras-Erk1/2/MAPK pathways (Terada et al., 2014). However, despite all this evidence, further research is required to understand the mechanism through which DEX disrupts neurotrophin signaling and Trk-mediated neurite outgrowth.

Over the last decade, evidence of an emerging signaling crosstalk between RTK and GRs have been reported, ranging from modulation of receptor levels to transcriptional induction of several protein regulators that function at multiple levels of the signaling cascade and at different time-points after receptor engagement (Shi and Mocchetti, 2000; Lauriola et al., 2014; D’Uva and Lauriola, 2016). It has been established that GR exploits a feedback module that restricts EGF receptor (EGFR) tyrosine kinase signaling through a mechanism entailing suppression of EGFR’s positive feedback loops and simultaneous triggering of negative feedback loops that normally restrain EGFR. Interestingly, the inhibitory effect of DEX was associated with shorter Erk1/2 activation, probably due to simultaneous induction of negative-feedback regulators of EGFR-induced Erk/MAPK signaling pathway, such as Sprouty4, Mig6 and the Dual-Specificity (Thr/Tyr) Phosphatase 1 (DUSP1; Lauriola et al., 2014). Thus, the same regulatory molecules induced by DEX represent negative-feedback regulators of EGF receptor signaling.

In support of a possible suppressive cross-talk between DEX and TrkA, it has been demonstrated that NGF signaling upregulates Erk-specific dual-specificity phosphatases, DUSPs, thereby leading to reduced Erk1/2 phosphorylation and constituting a negative-feedback loop for the prevention of axon overgrowth (Camps et al., 1998; Peinado-Ramon et al., 1998; Finelli et al., 2013). Furthermore, we have previously shown that *Sprouty4* is also a TrkA-induced gene that restricts Erk/MAPK pathway and neuronal differentiation of PC12 cells and primary sensory neurons in response to NGF (Alsina et al., 2012a). In this regard, it is important to understand the mechanism through which GC control neurotrophin signaling and Trk-mediated neuronal differentiation in different neuronal contexts. DUSP1 and Sprouty4 function as negative feedback regulators of receptor tyrosine kinase signaling. However, they do so by inhibiting the Ras-Erk1/2 pathway at different levels of the cascade, with Sprouty4 acting upstream than DUSP, probably at the level of Raf1 (Alsina et al., 2012a). Taking this into consideration, here we proposed to explore whether Sprouty4 could be mediating the negative effects of DEX on neurotrophin function. Interestingly, members of the Sprouty family, such as Sprouty2 and 4 have been observed in the cytoplasm and nucleus of hippocampal neurons and concentrated in dendritic and axonal growth cones. Moreover, downregulation of Sprouty2 and 4 strongly promoted axonal growth of cultured hippocampal neurons (Hausott et al., 2012).

Increasing evidence supports an essential role of the Erk/MAPK pathway in the pathogenesis and treatment of depression. For instance, a downregulated level of Erk phosphorylation was observed in the prefrontal cortex and hippocampus of animals exposed to chronic GC administration (Wang and Mao, 2019).

On the other hand, several studies have associated BDNF/TrkB signaling with depression and antidepressant drug action (Saarelainen et al., 2003; Bjorkholm and Monteggia, 2016). Antidepressant drugs normalize the BDNF-MAPK-CREB pathway in the hippocampus and thereby ameliorate depressive symptoms (Schmidt and Duman, 2010; Bjorkholm and Monteggia, 2016; Casarotto et al., 2021). Although the BDNF-MAPK-CREB pathway plays a key role in the pathophysiology and treatment of depression, the molecular mechanisms through which GC induces adaptive changes in this signaling cascade are not fully understood.

In view of this important evidence, we decided to investigate the role played by the negative feedback loop regulator of the Erk1/2/MAPK pathway, Sprouty4, in the mechanism of signaling crosstalk between DEX and neurotrophins.

## Materials and methods

### Ethics statement

Animal experiments were approved by the Institutional Animal Care and Ethics Committee of the School of Medicine (CICUAL-UBA), ethical permit number: 619/2021.

### Cell lines, recombinant proteins, and inhibitors

PC12 cells were grown in DMEM supplemented with 5% horse serum and 10% FBS (Invitrogen, MA, United States) as previously described (Ferrero Restelli et al., 2020). MN1 cells were cultured in DMEM supplemented with 10% FBS, 10 mM HEPES (Invitrogen) as previously described (Ledda et al., 2008; Alsina et al., 2016). NGF was purchased from Promega, DEX and the GC receptor inhibitor RU486 (2 μM) were obtained from Sigma (St. Louis, Missouri, United States).

### RT-PCR

The expression of *Sprouty4*, *TrkA*, and TATA box-binding protein (*Tbp*) mRNAs were analyzed by semiquantitative PCR from total RNA isolated from cultured PC12 cells or primary hippocampal neurons using NucleoSpin extraction kit (Macherey-Nagel, D*ü*ren, Germany). cDNA was synthesized using M-MLV Reverse Transcriptase (Invitrogen) and oligodT (Macrogen, Seoul, Korea). The cDNA was amplified using the following primer sets: *Sprouty4*: forward 5′-GCC CATTGA CCA GAT GAA GAC-3′; reverse 5′-TCC AGT GGCTTA CAG TGG AC-3′; *TrkA*: forward 5′-CTG CAA GGA CAA ACA GAA CAC-3′; reverse 5′-GTG GTT GGC TTC GTC TGA GTA-3′ and *Tbp*: forward 5′-GGGGAGCTGTGATGTGAAGT-3′, reverse 5′-CCAGGAAATAATTCTGGCTCA-3′.

For semiquantitative PCR analysis, agarose gel bands were quantified using Gel-Pro Analyzer software.

### Cell transfection and plasmids

MN1 cells were transfected with Polyethylenimine-PEI (Polysciences, PA, United States). PC12 cells were transfected using X-treme GENE (Roche) following the manufacturer’s instructions. PC12-Myc-Sprouty4-Y53A pool was generated by transfection and selection with neomycin.

Transient transfection of rat primary hippocampal neurons was performed using Lipofectamine 2000 (Invitrogen) in 300 μL of Neurobasal medium containing 1 μg of total plasmid DNA per well in 24-well plates containing 1.2 × 10^5^ cells/well.

For downregulation experiments, PC12 cells and hippocampal neurons were transfected with 1 μg of control-shRNA or *Sprouty4-shRNA* constructs expressing GFP protein. Control-shRNA-GFP and *Sprouty4-shRNA*-GFP constructs were purchased from OriGene (Rockville, MD, United States) and used according to manufacturer’s protocols. The retroviral vector pGFP-V-RS was used for expression of shRNA targeting rat *Sprouty*4. The sequence of the *Sprouty4-shRNA* is 5′-GTG CAA GGT ATC TTC TAC CAC TGT ACT AA-3′, and corresponds to nucleotides 610–638 of rat *Sprouty*4. The specificity of Sprouty4 knockdown was previously confirmed by real-time PCR and immunoblotting (Alsina et al., 2012a).

For overexpression experiments, PC12 cells were co-transfected with Myc-Sprouty4-Y53A (0.9 μg) construct and GFP expression vector (0.1 μg) per well in 24 well plates containing 0.6–0.75 × 10^5^ cells/well. Myc-Sprouty4-Y53A construct was kindly provided by Dr. Akihiko Yoshimura (Kyushu University, Fukuoka, Japan).

### Primary culture of hippocampal neurons

Rat (Sprague Dawley) were acquired from Instituto de Biología Celular y Neurociencia, CONICET-UBA and hippocampal neurons from embryonic day (E) 17.5 were dissociated by trituration and cultured in Neurobasal medium (Gibco) supplemented with B27 (Gibco), penicillin, streptomycin, and GlutaMax (Gibco) as previously described (Alsina et al., 2016).

For transfection, hippocampal neurons were seeded in 24-well dishes at a density of 1.2 × 10^5^ cells/well. Cultures were grown for 3 days *in vitro* (DIVs) before transfection.

### Total cell lysates and western blotting

Cells were lysed at 4°C in buffer containing 0.5% Triton X-100 plus protease and phosphatase inhibitors (sodium orthovanadate 1 mM and sodium fluoride 20 mM). Protein lysates were clarified by centrifugation and analyzed by Western blotting as previously described (Ledda et al., 2008). The blots were scanned in a Storm 845 PhosphorImager (GE Healthcare Life Sciences), and quantifications were done with ImageQuant software (Molecular Dynamics). Briefly, lanes were manually adjusted and an intensity profile was automatically generated for each lane. Band limits were automatically detected and background was subtracted using a rolling ball method correction. Band intensity was then measured as area under the curve.

The primary antibodies were obtained from various sources as follows: Anti-Myc tag (1:2,000; A190-105A) was from Bethyl; anti-phospho Erk1/2 MAPK (p44/p42; 1:3,000; #9101), anti-Erk1/2 MAPK (p44/p42; 1:5,000; #4695), and anti-p75^NTR^ (1:2,000; D4B3-#8,238) were from Cell Signaling Technology; anti-Sprouty4 (1/800; H-100) was from Santa Cruz Biotechnology; and anti-α-Tubulin (1:6,000; T9026–DM1A) was from Sigma.

### Immunofluorescence

For phospho-Erk1/2 (pErk1/2) assays, hippocampal primary cultures or PC12 cells were fixed with 4% PFA containing phosphatase inhibitors (sodium orthovanadate 1 mM and sodium fluoride 20 mM), permeabilized with 0.25% Triton X-100, blocked with 10% donkey serum (Jackson ImmunoResearch), and then incubated with different primary antibodies. The antibodies used in this section included: anti-GFP (1/1,000) from Invitrogen and anti-pErk1/2 (p44/p42; 1:500; #9101 l) from Cell Signaling Technology. Secondary antibodies were from Jackson ImmunoResearch (1/300).

### Image acquisition and quantification

Images for quantification of pErk1/2 MAPK activation were obtained using an Olympus IX-81 20x objective. Images were acquired using the same settings with no saturation and no bleed through and minimized noise at a resolution of 1,360 × 1,024 pixels. Images were analyzed using the Fiji ImageJ software and Corrected Total Cell Fluorescence (CTCF) was calculated for each individual cell using a hand-drawn ROI. CTCF is calculated as the integrated density of the selected cell minus the area of the selected cell times the mean fluorescence of background readings [CTCF = Integrated Density of ROI − (Area of ROI × Mean fluorescence of background)].

### PC12 neurite outgrowth and hippocampal neurite outgrowth assays

PC12 cells were transfected with control-shRNA or *Sprouty*4-shRNA, or overexpressing a dominant negative mutant Myc-Sprouty4-Y53A or empty vector together with a GFP plasmid.

Cells were then plated on 24-well plates and pretreated or not with dexamethasone (DEX; Sigma) 1 μM for 16 h in serum-free medium. Then, medium was changed by fresh serum-free DMEM containing NGF (50 ng/mL). After 24 h, cells were fixed with 4% paraformaldehyde (PFA). The percentage of cells bearing neurites longer than one or two cell bodies diameter was quantified as we previously described (Ferrero Restelli et al., 2020).

Hippocampal cultures were grown and transfected at DIV3 with either control-shRNA-GFP or *Sprouty*4-shRNA-GFP constructs. Twenty four hours after transfection (at DIV4) the cultures were pretreated with dexamethasone (DEX, 10 μM; Kumamaru et al., 2008). Then, at DIV5, BDNF (50 ng/mL) was also applied in the presence of DEX. Forty-eight hours after BDNF addition, the neuronal cells were fixed.

The number of primary neurites and branching points were determined on shRNA transfected (GFP-positive) neuronal cells that displayed a pyramidal-shaped morphology bearing a thick main dendrite and several thin dendrites. Neuritic processes <5 μm in length were not counted (Alsina et al., 2016).

In all cases images were obtained using an Olympus IX-81 inverted microscope with a 20× objective. Images were acquired using the same settings with no saturation and no bleed through and minimized noise at a resolution of 1,360 × 1,024 pixels.

### Statistical analysis

Data are reported as mean ± s.e.m. or s.d. as indicated, and significance was accepted at *p* < 0.05. Studentʼs *t*-test or ANOVA followed by Tukey or Dunnett *post-hoc* tests were performed using GraphPad.

## Results

### Dexamethasone induces the expression of *Sprouty4 mRNA*

The synthetic glucocorticoid dexamethasone (DEX) has been described as a potent inhibitor of the biological effects and downstream signaling pathways triggered by growth factors and neurotrophins (Unsicker et al., 1978; Kumamaru et al., 2008; Kawashima et al., 2010; Kumamaru et al., 2011; Lauriola et al., 2014; Terada et al., 2014; Matsushima et al., 2019). It has been shown that DEX enhances the EGF-induced expression of different negative feedback loop regulators of the Ras-Erk1/2/MAPK pathway (Lauriola et al., 2014; Gelfo et al., 2019).

Previously, we have reported that Sprouty4 is a NGF-induced gene that retro-regulates TrkA-mediated Erk1/2 activation to restricts neurite outgrowth (Alsina et al., 2012a). Thus, based on all this evidence, we decided to examine whether the inhibitory effect of DEX on neurotrophic factor signaling might involve Sprouty4. In order to analyze whether DEX induces the expression of Sprouty4 in neuronal cells, we evaluated the kinetics of mRNA expression of this negative regulator upon DEX or NGF treatment by semiquantitative RT-PCR. For these assays, we used the neuroblast-like PC12 cell line, which provide a robust cellular model to study NGF function, because they express both p75^NTR^ and TrkA neurotrophin receptors and differentiate into a neuronal-type resembling sympathetic neurons in response to NGF.

**Figure 1:**
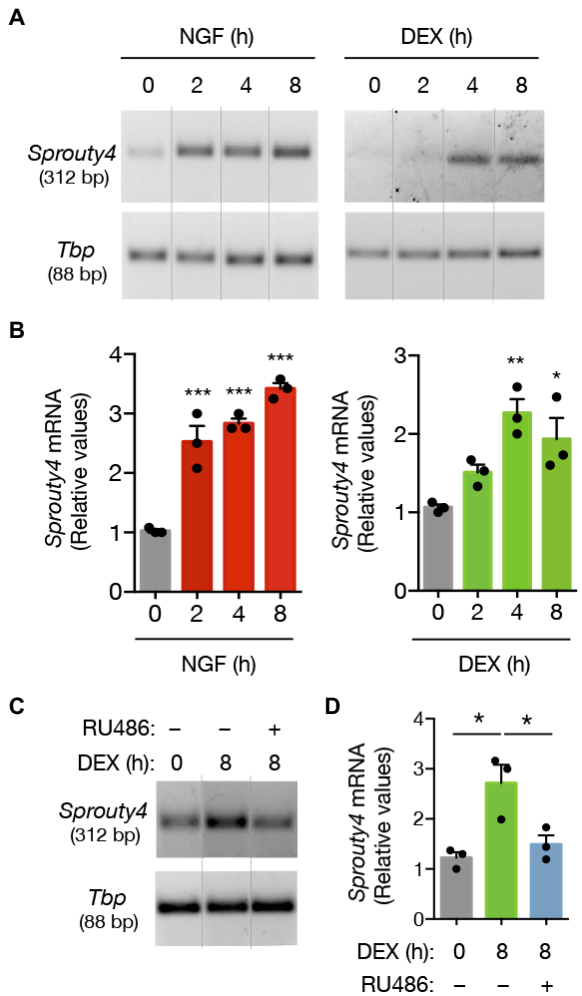
DEX promotes the expression of different negative feedback regulators of NGF-induced Ras-Erk1/2/MAPK pathway. **(A)** Semiquantitative RT-PCR analysis of *Sprouty4* mRNA. PC12 cells were treated with DEX (1 μM) or NGF (50 ng/mL) for different time-points. For each molecule, the PCR amplification product size is indicated in base pairs (bp). **(B)** Bar graphs showing the induction profiles of *Sprouty4* mRNA at different time points upon DEX or NGF stimulation. The levels of mRNA were normalized to the expression of the housekeeping gene *Tbp*. Values are presented as relative to untreated control group (t = 0 h DEX or NGF). The results are shown as mean ± s.e.m. of *n* = 3 independent assays. ^*^p < 0.05, ^**^p < 0.01, ^***^p < 0.001 by one-way ANOVA followed by Dunnett’s-test. **(C)** Semiquantitative RT-PCR analysis of *Sprouty4* mRNA. For this assay, PC12 cells were pretreated or not with the GR inhibitor RU486 (2 μM) and then stimulated with DEX (1 μM) for 8 h. **(D)** Bar graph showing the blockade of DEX-induced expression of *Sprouty4* mRNA by the pretreatment with the GR antagonist RU486. The levels of mRNA were normalized to the expression of the housekeeping gene *Tbp*. Values are presented as relative to untreated control group (t = 0 h DEX, gray bar). ^*^p < 0.05 by one-way ANOVA followed by Tukey’s multiple comparisons test. Dotted lines in panels **A** and **C** indicate representative bands cut from the same PCR gel image.

Notably, our findings showed that while NGF induces a significant increase in the levels of *Sprouty4* mRNA expression, treatment of PC12 cells with DEX also promotes a significant increase (~2-fold) in the expression of this molecule, which last for at least 8 h post-stimulation (Figures 1A,B). Thus, Sprouty4 induction by DEX and NGF indicates that Sprouty4 might be mediating the signaling crosstalk between GC and NGF. The pretreatment with the GR antagonist RU486 abrogated the DEX-induced expression of *Sprouty4* mRNA (Figure 1C), indicating that DEX exerts this effect acting through GR.

Thus, our results uncover a transcriptional crosstalk between DEX and NGF, which involves the activation of the endogenous negative feedback regulator of the TrkA-promoted Ras-Erk1/2 signaling pathway, Sprouty4.

### Sprouty4 mediates the inhibitory effect of GC on TrkA-mediated Erk1/2 signaling

Based on the fact that DEX has been described as a potent inhibitor of the biological effects of neurotrophins, and in our previous observation indicating that Sprouty4, is induced by DEX in neuronal cells, we proposed that the induction of Sprouty4 by DEX pretreatment could translate into a strong inhibition of the NGF/TrkA downstream signaling pathway.

**Figure 2:**
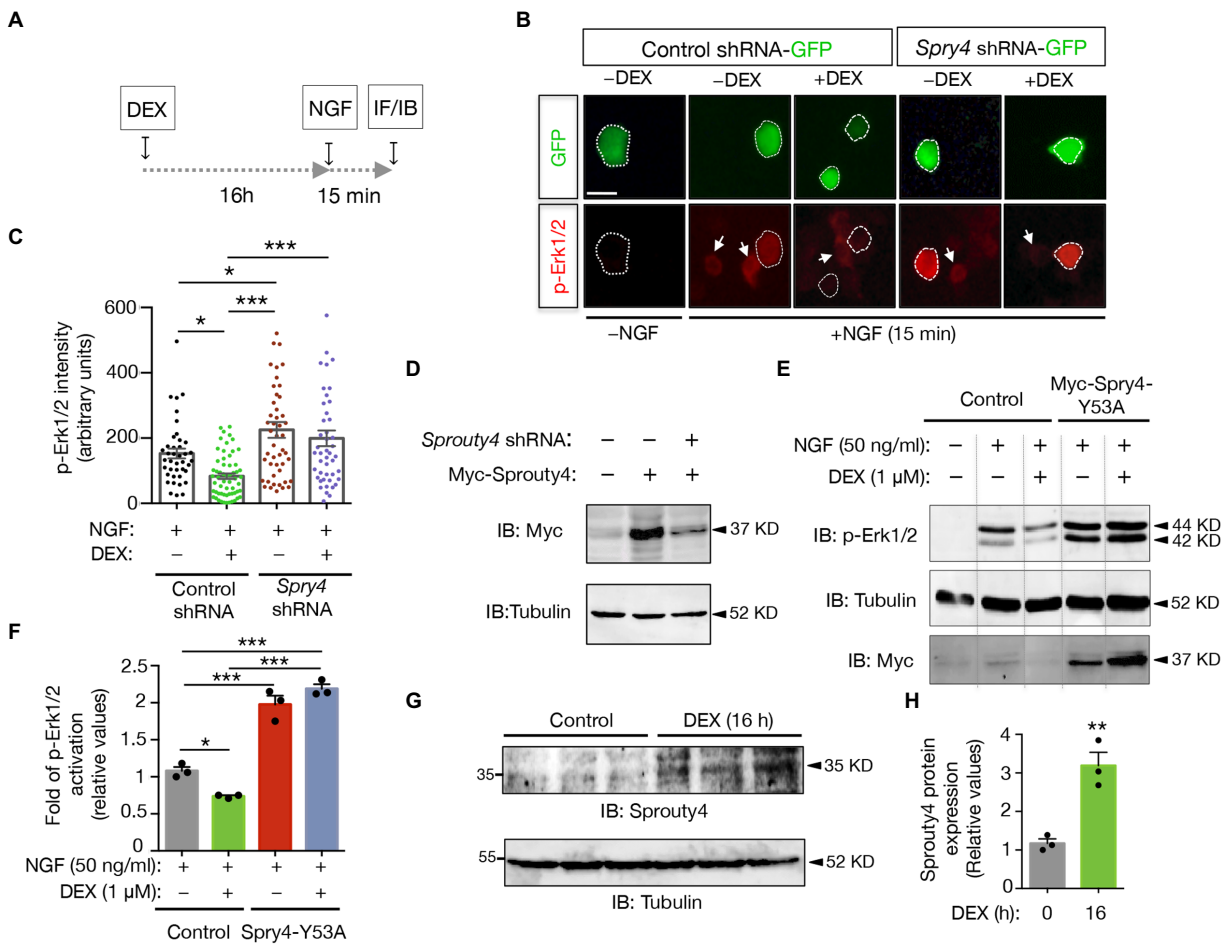
Sprouty4 mediates the inhibitory effect of DEX on NGF-induced Erk1/2 signaling. **(A)** Experimental schemes used to measure Erk1/2 phosphorylation in response to DEX and NGF treatments. Panels **B** and **C** show the evaluation of Erk1/2 activation by immunofluorescence (IF) and panels **E** and **F** by IB. **(B)** Photomicrographs show PC12 cells transfected with control-shRNA-GFP or *Sprouty4*-shRNA-GFP plasmids. Cells were pretreated or not with DEX (1 μM) for 16 h, stimulated for 15 min with NGF (50 ng/mL) and then fixed with 4% PFA. Activation of Erk1/2 was assessed by immunofluorescence (IF) of phospho Erk1/2 (p-Erk1/2) on GFP-positive cells labeled with dotted lines. Arrows indicate neighboring non-transfected cells. Scale bar, 15 μm. **(C)** Graph showing individual values of p-Erk1/2 activation expressed as arbitrary units. 40–60 cells/experimental condition were evaluated. Data are mean ± s.e.m. of *n* = 3 independent assays. ^*^p < 0.05, ^***^p < 0.001 by one-way ANOVA followed by Tukey’s multiple comparisons test. **(D)** Sprouty4 protein levels were analyzed by immunoblotting (IB) in MN1 cells transfected with control or Sprouty4-shRNA vector together with Myc-tagged Sprouty4. Tubulin is shown as loading control. **(E)** Representative IBs showing Erk1/2 activation (p-Erk1/2) in PC12 cell extracts transfected with control or Myc-tagged Sprouty4-Y53A (Myc-Spry4-Y53A) constructs. Cells were pretreated or not with DEX (1 μM) for 16 h, and then stimulated for 15 min with NGF (50 ng/mL). Reprobing of the same blot with anti-Tubulin and anti-Myc are shown. Dotted lines indicate representative bands cut from the same immunoblot image. **(F)** Quantification of the fold of p-Erk1/2 activation relative to control cells only treated with NGF for 15 min (gray bar). Values are mean ± SD of *n* = 3 independent assays. ^*^p < 0.05, ^***^p < 0.001 by one-way ANOVA followed by Tukey’s-test. **(G)** Immunoblot showing increased Sprouty4 protein levels upon 16 h of DEX treatment. Reprobing of the same filter with anti-Tubulin is shown as loading control. **(H)** Quantification of the levels of Sprouty4 expression in control and DEX-treated cells for 16 h. Values are expressed relative to the untreated control group. Bars represent mean ± SD of *n* = 3 independent samples. ^**^p < 0.01 by Studentʼs *t*-test.

In order to analyze whether the DEX-induced expression of Sprouty4 could be responsible of the DEX inhibitory effects on neurotrophin signaling, we decided to analyze NGF-induced Erk/MAPK activity in PC12 cells transfected with either *Sprouty4*-shRNA-GFP or control-shRNA-GFP and pretreated with DEX (Figure 2A). For this assay, we used a previously validated plasmid-based shRNA interference system to knockdown rat *Sprouty*4 expression (Alsina et al., 2012a). In these assays, activation of Erk1/2 was evaluated in GFP-positive transfected cells by immunofluorescence using a specific anti-phospho MAPK/Erk1/2 (Thr202/Tyr204) antibody. Interestingly, whereas in control cells, the pretreatment with DEX for 16 h, significantly inhibited NGF-mediated Erk1/2 activation, knockdown of *Sprouty4* expression abrogated the inhibitory effect of DEX on NGF-induced Erk1/2 phosphorylation (Figures 2B,C). The efficiency of the shRNA construct was additionally controlled by immunoblotting. As shown in Figure 2D, a substantial reduction of ectopically expressed Myc-tagged Sprouty4 protein was detected in lysates obtained from *Sprouty4*-shRNA MN1 transfected cells.

Next, in order to confirm this result indicating that downregulation of Sprouty4 expression abrogates the inhibitory effect of GC on NGF-induced MAPK activation, we performed an additional experiment using a mutated form of Sprouty4 in which a conserved tyrosine residue was replaced by alanine (Sprouty4-Y53A). This mutation generates a dominant negative form of Sprouty4 that enhances the trophic factor-dependent Erk1/2/MAPK activation (Sasaki et al., 2001). To this end, PC12 cells transfected with either Sprouty4-Y53A mutant or control plasmid were pretreated or not with DEX, then stimulated with NGF, and Erk1/2/MAPK activity was analyzed by immunoblot. This assay showed that pretreatment with DEX significantly inhibits NGF-induced Erk1/2 phosphorylation in control cells, but failed to reduce NGF-induced Erk1/2 activation in extracts of PC12 cells transfected with the non-functional Sprouty4-Y53A mutant (Figures 2E,F). Although phospho Erk1/2 levels were normalized to the housekeeping Tubulin, we have also controlled that neither DEX treatment nor Sprouty4 Y53 mutant expression affected total Erk1/2/MAPK levels among these experimental groups (Supplementary Figure S1).

To confirm that the induction of *Sprouty4* mRNA by GC, results in a significant increase of protein levels upon GC treatment, we controlled the expression of Sprouty4 protein by immunoblot in PC12 cells treated with DEX for 16 h. Consistent with the induction of *Sprouty4* mRNA (Figure 1), immunoblotting assay confirmed a significant increase of the Sprouty4 protein levels in PC12 cells treated with this GC for 16 h, the time in which we stimulated the cells with NGF and performed the analysis of Erk1/2/MAPK activity (Figures 2G,H).

Together, these findings indicate that Sprouty4 mediates the suppressive effect of DEX on NGF-induced Erk1/2/MAPK activation in neuronal cells.

### Sprouty4 mediates the inhibitory effect of GC on NGF-induced neurite outgrowth

**Figure 3:**
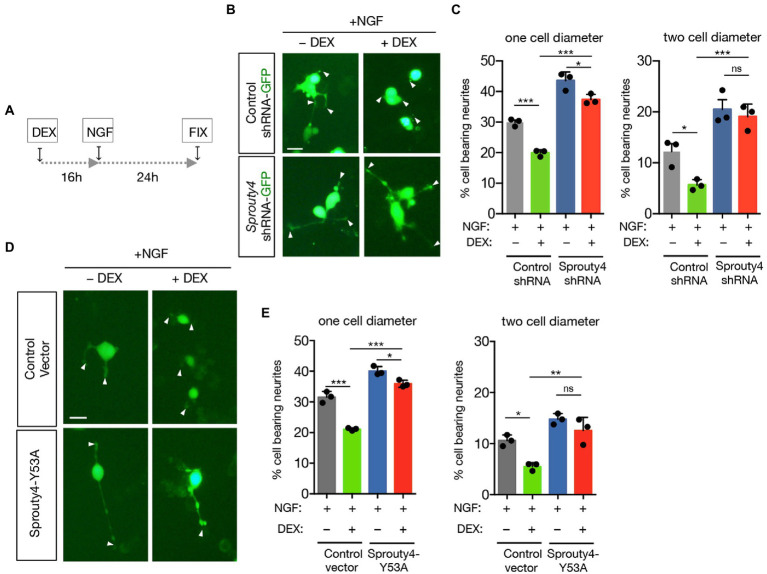
Knockdown of *Sprouty4* limits the inhibitory effect of DEX on neurite outgrowth induced by NGF. **(A)** Experimental schedule of DEX and NGF treatment for PC12 neurite outgrowth. Fix means fixation. **(B)** Photomicrographs show PC12 cells transfected with control-shRNA-GFP or *Sprouty4*-shRNA-GFP constructs and pretreated or not with DEX (1 μM) and stimulated for 24 h with NGF (50 ng/mL). Arrowheads indicate neurite tips. Scale bar, 20 μm. **(C)** Graph shows neurite outgrowth of PC12 cells transfected with control-shRNA or *Sprouty4*-shRNA plasmids and pretreated or not with DEX and stimulated for 24 h with NGF. Quantification of the % of GFP-positive cells bearing neurites longer than one or two cell body diameter in the different experimental groups. For each experimental condition we quantified 120 images with approximately 15 to 20 cells per picture. Data are mean ± SD of *n* = 3 independent experiments. ^*^p < 0.05, ^***^p < 0.001 by one-way ANOVA followed by Tukey’s multiple comparisons test. ns, not significant. **(D)** Photomicrographs show PC12 cells transfected with control or Sprouty4-Y53A constructs together with a GFP-expression vector and pretreated or not with DEX (1 μM) and then stimulated for 24 h with NGF (50 ng/mL). Arrowheads indicate neurite tips. Scale bar, 20 μm. **(E)** Graph shows neurite outgrowth of PC12 cells transfected with control or Sprouty4-Y53A constructs together with a GFP-expression vector and pretreated or not with DEX (1 μM) and then stimulated for 24 h with NGF (50 ng/mL). Quantification of the % of GFP-positive cells bearing neurites longer than one or two cell body diameters in the different experimental groups. For each experimental condition we quantified 120 images with approximately 15 to 20 cells per picture. Data are mean ± SD of *n* = 3 independent experiments. ^*^p < 0.05, ^**^p < 0.01, and ^***^p < 0.001 by one-way ANOVA followed by Tukey’s multiple comparisons test. ns, not significant.

Next, we decided to explore the biological consequences of this GR to TrkA receptor crosstalk. As it has been described that GC pretreatment inhibits the neurite outgrowth induced by NGF on PC12 cells, we decided to analyze whether knockdown of *Sprouty4* expression, abrogates the effect of GC on NGF-induced neurite outgrowth. In order to perform this analysis, PC12 cells were transfected with control-shRNA-GFP or *Sprouty4*-shRNA-GFP vectors and the ability of DEX to block neurite outgrowth in response to NGF was evaluated. Interestingly, whereas in control cells the pretreatment with DEX significantly inhibited NGF-promoted neurite outgrowth, knockdown of *Sprouty4* expression prevented the inhibitory effect of DEX on neurite outgrowth induced by NGF (Figures 3A–C). Interestingly, this inhibitory effect of DEX on NGF-mediated neurite outgrowth observed in control cells was abolished by downregulating *Sprouty4* expression in PC12 cells extending neurites longer than two cell body diameter. Additionally, the overexpression of a dominant negative form of Sprouty4 (Y53A), which fails to block Erk1/2/MAPK pathway (Alsina et al., 2012a), rescued the inhibitory effect of DEX on neurite outgrowth induced by NGF (Figures 3D,E).

No differences were detected in *TrkA* mRNA and p75^NTR^ protein levels in PC12 cells treated with DEX for 16 h (Supplementary Figure S2), indicating that the mechanism through which DEX abrogated NGF function does not involve changes in the expression of neurotrophin receptors.

Taken together, these findings indicate that blocking the function of *Sprouty4*, significantly limited the ability of DEX to inhibit TrkA to Erk1/2 signaling and neurite outgrowth in response to NGF.

### Knockdown of *Sprouty4* abrogates the inhibitory effects of DEX on BDNF-induced morphological differentiation and Erk1/2 phosphorylation in hippocampal neurons

It has been demonstrated that pretreatment with DEX prevents BDNF-induced up-regulation of both the number of primary dendrites and maturation of synaptic function in developing hippocampal neurons by reducing Erk1/2/MAPK activity (Kumamaru et al., 2008; Kawashima et al., 2010). As Sprouty2 and 4 are expressed in hippocampal developing neurons and their downregulation by shRNAs strongly promotes neurite outgrowth (Gross et al., 2007; Hausott et al., 2012), we decided to explore whether Sprouty proteins might also play a crucial role in the control of BDNF function by DEX in primary cultures of hippocampal neurons.

**Figure 4:**
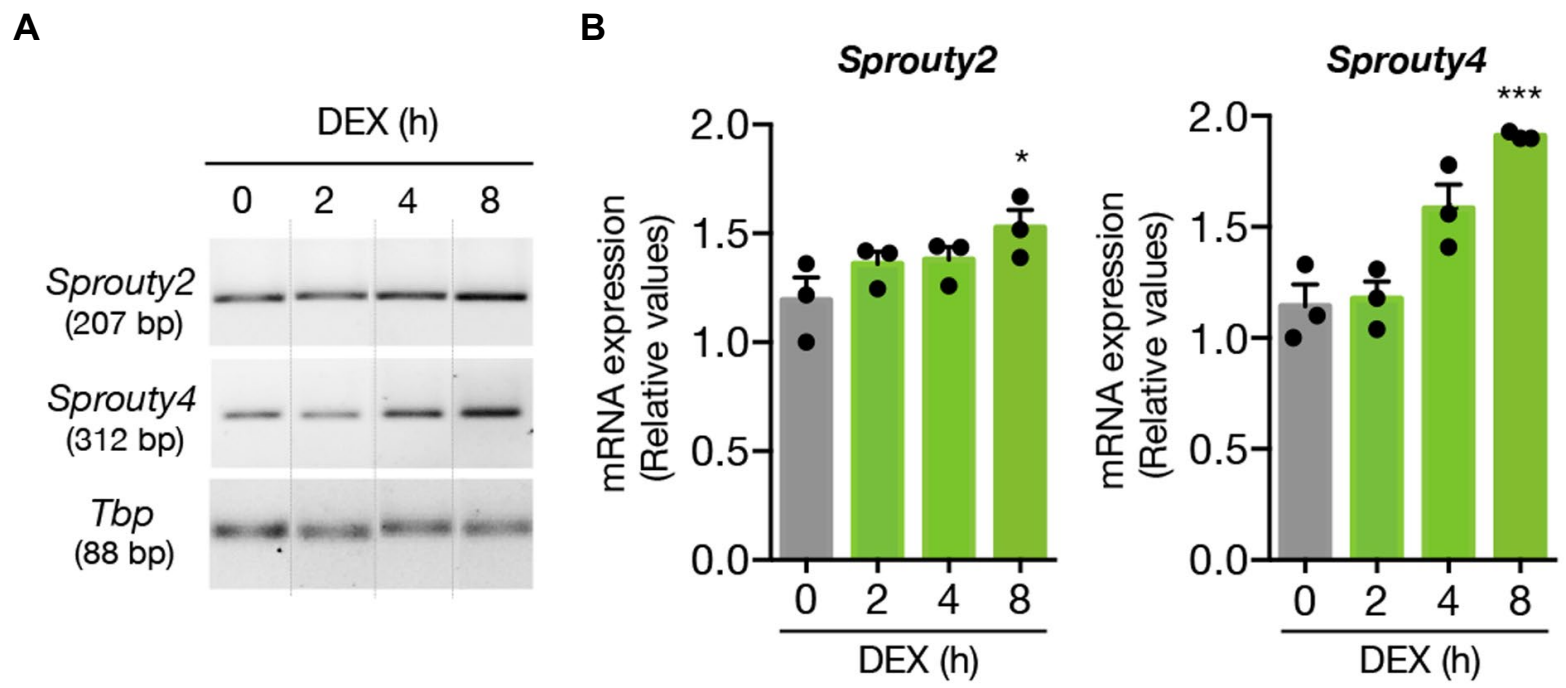
DEX promotes the expression of *Sprouty2* and *Sprouty4* mRNAs in hippocampal primary neurons. **(A)** Semiquantitative RT-PCR analysis of *Sprouty2* and *Sprouty4* mRNA expression. Hippocampal primary cultures were treated with DEX (1 μM) for different time-points. For each molecule, the PCR amplification product size is indicated in base pairs (bp). Dotted lines indicate representative bands cut from the same PCR gel image. **(B)** Bar graphs showing the induction profiles of *Sprouty2* and *Sprouty4* mRNAs at different time points upon DEX stimulation. The levels of mRNAs were normalized to the expression of the housekeeping gene *Tbp*. Values are presented as relative to the untreated control group (t = 0 h DEX, gray bar). The results are shown as mean ± s.e.m. of *n* = 3 independent assays. ^*^p < 0.05, ^***^p < 0.001, by one-way ANOVA followed by Dunnett’s-test.

First, we examined whether DEX treatment might enhance the expression of Sprouty2 and Sprouty4 in hippocampal neurons. By using semiquantitative RT-PCR we evaluated the kinetics of mRNA expression of *Sprouty2* and *Sprouty4* upon DEX treatment. Our findings showed that while DEX treatment promoted a slight but significant increase in Sprouty2 expression levels, the same treatment triggered a more robust and significant increase in *Sprouty4* mRNA expression, which last for at least 8 h post-stimulation (Figure 4).

**Figure 5:**
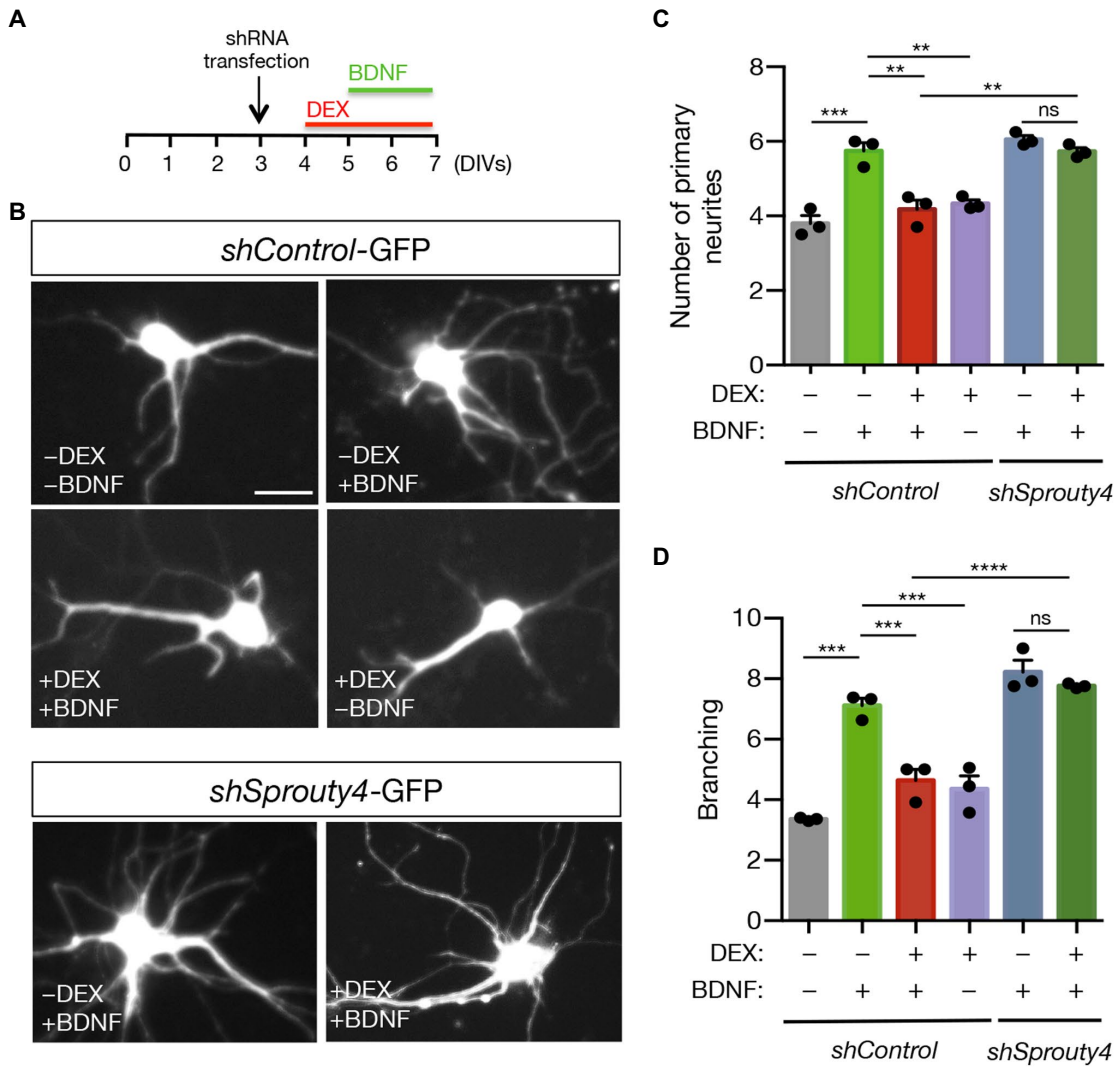
Knockdown of *Sprouty4* abrogates the inhibitory effect of DEX on the morphological differentiation induced by BDNF in hippocampal neurons. **(A)** Experimental scheme of control and *Sprouty4* shRNA transfected neurons treated with DEX and/or BDNF for the indicated days *in vitro* (DIV). **(B)** Representative images of rat hippocampal neurons transfected with control or *Sprouty4* shRNA vectors and treated or not with DEX and/or BDNF as indicated in **(A)**. Scale bar, 20 μm. **(C,D)** Quantification of primary dendrites **(C)** and total dendritic branching **(D)** of hippocampal neurons transfected with either control or *Sprouty4*-shRNA-GFP vector and then treated with DEX and/or BDNF as indicated in **(A)**. A total of 36–45 neurons per experimental condition were analyzed. The results are shown as mean ± s.e.m. of *n* = 3 independent assays. ^**^p < 0.01, ^***^p < 0.001, ^****^p < 0.0001 by one-way ANOVA followed by Tukey’s multiple comparisons test. ns, not significant.

Based on this result, we decided to further explore whether Sprouty4 mediates the functional consequences of GR to TrkB receptor crosstalk by transfecting hippocampal neurons with control-shRNA-GFP or *Sprouty4*-shRNA-GFP vectors and testing the ability of DEX to block primary neurite formation and proximal dendrite branching in response to BDNF. Interestingly, whereas the pretreatment with DEX significantly reduces both primary neurites and dendrite branching effects of BDNF, knockdown of *Sprouty4* expression abrogates the inhibitory effect of DEX on these morphological parameters induced by BDNF (Figures 5A–D). Together, these assays reveal that Sprouty4 also mediates the inhibitory effects of DEX on the morphological differentiation of hippocampal neurons induced by BDNF.

**Figure 6:**
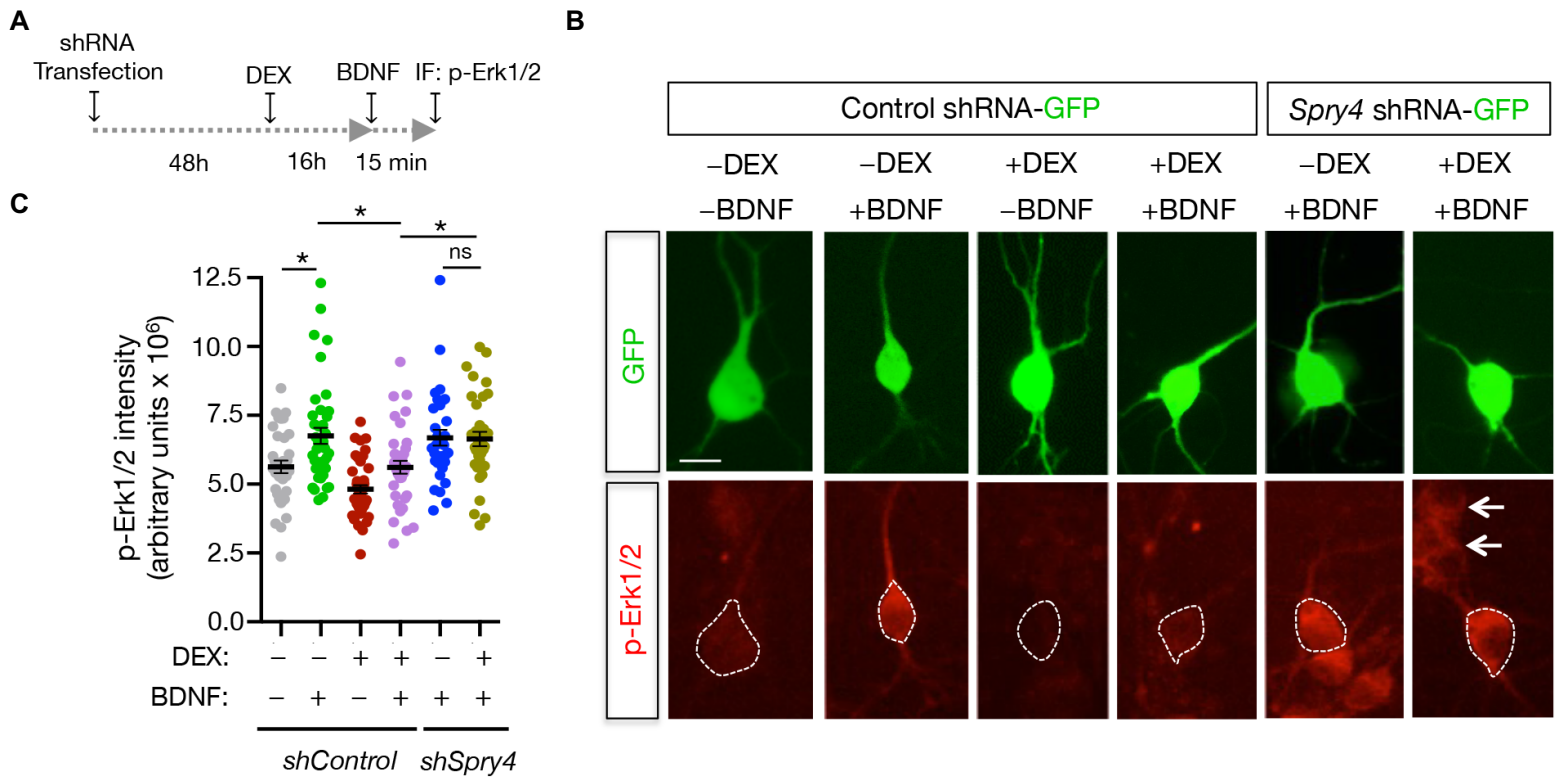
Sprouty4 mediates the inhibitory effect of DEX on BDNF-induced Erk1/2 phosphorylation in hippocampal neurons. **(A)** Experimental scheme used to measure Erk1/2 phosphorylation (p-Erk1/2) in response to DEX and BDNF treatments. Hippocampal neurons were transfected at DIV3 with control-shRNA-GFP or *Sprouty4*-shRNA-GFP plasmids. **(B)** Photomicrographs show hippocampal neurons transfected with control-shRNA-GFP or *Sprouty4*-shRNA-GFP plasmids. Neurons were pretreated or not with DEX for 16 h, stimulated for 15 min with BDNF (25 ng/mL) and then fixed with 4% PFA containing phosphatase inhibitors. Activation of Erk1/2 was assessed by immunofluorescence (IF) of phospho-Erk1/2 (p-Erk1/2) on GFP-positive cells labeled with dotted lines. Arrows indicate neighboring non-transfected cells. Scale bar, 20 μm. **(C)** Graph showing individual values of p-Erk1/2 activation expressed as arbitrary units. A total of 33–43 individual neurons per experimental condition were analyzed. Data are mean ± s.e.m. of *n* = 3 independent assays. ^*^p < 0.05 by one-way ANOVA followed by Tukey’s multiple comparisons test.

BDNF-promoted increases in primary neurite formation and dendrite branching occur *via* activation of the MAPK/CREB pathway (Kwon et al., 2011). Since Sprouty4 mediates the inhibitory effect of DEX on BDNF-induced morphological differentiation in developing hippocampal neurons, we decided to analyze whether Sprouty4 downregulation abrogates the inhibitory effect of GC on BDNF-induced Erk1/2/MAPK activation. For this purpose, hippocampal primary neurons were transfected with either control-shRNA-GFP or *Sprouty4*-shRNA-GFP vectors and then pretreated or not with DEX, stimulated with BDNF and fixed with PFA as indicated in Figure 6A. BDNF-induced activation of Erk1/2 was evaluated in GFP-positive transfected neurons by immunofluorescence using a specific anti-phospho Erk1/2/MAPK (Thr202/Tyr204) antibody. Interestingly, whereas in control neurons, the pretreatment with DEX significantly inhibited BDNF-mediated Erk1/2 phosphorylation, knockdown of *Sprouty4* expression abrogated the inhibitory effect of DEX on BDNF-induced Erk1/2 phosphorylation (Figures 6B,C). Taken together, these findings also show that Sprouty4 mediates the inhibitory effects of DEX on the signaling and biological functions of BDNF investigated here in hippocampal neurons.

## Discussion

It has been established that chronic glucocorticoid (GC) exposure either by chronic stress or excessive GC treatment produces adverse effects for neuronal development and function, since it reduces neurite outgrowth, promotes dendritic branch atrophy and impairs hippocampal neurogenesis and synaptic plasticity (Magarinos et al., 1996; Wellman, 2001; Radley et al., 2004; Mayer et al., 2006; Radley et al., 2006; Kumamaru et al., 2008; Liston and Gan, 2011; Lucassen et al., 2015; Agasse et al., 2020).

Notably, sustained GC exposure also interferes with the biological functions triggered by NGF and BDNF in peripheral and central neurons, hence the adverse effects of GC on neuronal development may be due to a reduced neurotrophin signaling (Unsicker et al., 1978; Suri and Vaidya, 2013). In this regard, it has been demonstrated that GC prevents BDNF-induced dendritic growth and maturation of synaptic function in developing hippocampal and cortical neurons, by reducing activation of Erk1/2/MAPK pathway (Kumamaru et al., 2008; Numakawa et al., 2009; Kumamaru et al., 2011). Further insights into the crosstalk between GC and neurotrophins come from studies in PC12 cells showing that DEX prevents NGF signaling and TrkA-mediated neurite outgrowth (Terada et al., 2014). Although several studies have shown that GC treatment disrupts neurotrophin signaling and function, a better understanding of the molecular mediators underlying this inhibitory crosstalk is necessary.

**Figure 7:**
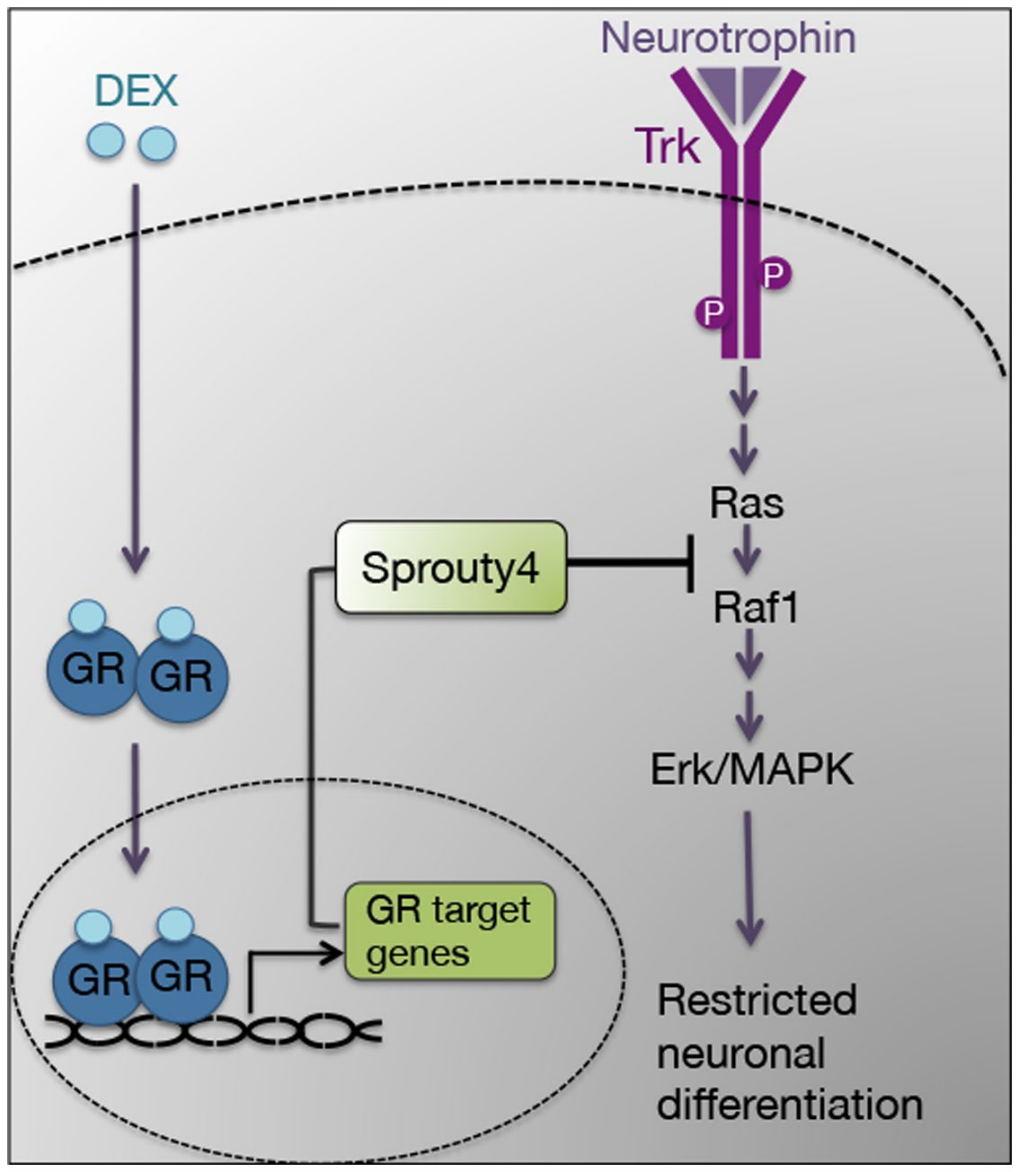
Model describing the proposed role of Sprouty4 as a signaling mediator of the inhibitory effect of DEX on neurotrophin-induced Erk1/2 pathway and neuronal differentiation.

In this study, we demonstrate that the negative regulator Sprouty4 mediates the inhibitory effect of DEX on neurotrophin function. Our findings show that the pretreatment with DEX induces the expression of Sprouty4, which specifically abrogates Ras/Erk1/2 signaling pathway in response to the neurotrophins NGF and BDNF in PC12 cells and hippocampal primary neurons, respectively (Figure 7). In PC12 cells, knockdown of *Sprouty4* rescues the inhibition of NGF-promoted neurite outgrowth and Erk1/2 phosphorylation induced by DEX. Moreover, the downregulation of Sprouty4 in hippocampal neurons blocks the inhibitory effects of DEX on the morphological changes induced by BDNF, such as dendrite formation and branching. Our results are consistent with previous studies showing that knockdown of *Sprouty*3 expression causes an excess of axonal branching in spinal cord motor neurons *in vivo* and negatively regulates signaling downstream of BDNF–TrkB (Panagiotaki et al., 2010).

In line with our findings, it has been reported that in different cell lines, GC inhibits EGF signaling by promoting the transcriptional induction of different negative feedback loop regulators of EGFR and Erk1/2/MAPK pathway such as Lrig1-3, Mig6, DUSP1 and Sprouty4 (Xu et al., 2005; Lauriola et al., 2014; Gelfo et al., 2019). More recently, it has been shown that DUSP1 and Sprouty1/4 are also induced in the lymphoblastic leukemia cell line SUP-B15 after 6 h of DEX treatment, indicating that these negative signaling regulators are part of a transcriptional program triggered by GC in different cellular contexts (Shah et al., 2021). Despite this evidence, the molecular mechanism through which DEX induces the expression of Sprouty4 is still unknown and requires additional investigation.

A previous study showed that chronic exposure of mature cortical neurons to DEX reduces the biological function of BDNF by suppressing miR-132 expression (Kawashima et al., 2010). Thus, in addition to facilitating the expression of negative regulators that normally restrain TrkB and Erk1/2 pathway, DEX also suppresses the BDNF-induced transcription of the positive feedback loop regulator of the Erk1/2 pathway miR-132. Unknown phosphatases that prevent Erk1/2 activation have been suggested as potential targets of the microRNA miR-132. Moreover, Kumamaru and colleagues have also found that chronic DEX treatment on developing cortical neurons suppresses BDNF-mediated activation of Erk1/2 through the inhibition of TrkB interaction with the Shp2 adapter protein (Kumamaru et al., 2011).

In addition to inhibit the TrkB-mediated Erk1/2 pathway, chronic glucocorticoid exposure can also regulate phospholipase C-γ (PLC-γ) activation in response to BDNF. Thus, through reducing the interaction of TrkB with cytosolic GC receptors, chronic exposure to GC weakens the activation of the PLC-γ/Ca2+ pathway and thus suppresses BDNF-induced glutamate release in cultured cortical neurons (Numakawa et al., 2009).

However, GC can also display positive effects for neuronal physiology. Thus, in a previous study, Jenneteau and colleagues reported that short-term DEX stimulation promoted neuronal survival by inducing the selective phosphorylation of TrkB and the activation of its downstream signaling effectors Akt, Erk1/2 and PLCγ through a mechanism that does not requires neither production nor co-stimulation with neurotrophins (Jeanneteau et al., 2008). This TrkB signaling activation was maximal between 2 and 4 h after DEX application and returned to basal levels at 6 h. Conversely, here we performed a longer GC pretreatment (16 h) followed by neurotrophin (NGF or BDNF) stimulation, to assess how pretreatment with DEX inhibits neurotrophin signaling and function instead to directly evaluate how short-term DEX treatment *per se* promotes Trk receptor activation, independently of neurotrophin stimulation.

Together, all this evidence shows that GC could either inhibit or promote neurotrophic function and signaling by acting through multiple mechanisms of action, which in turn depend mainly on the type of treatment (acute vs. chronic), the neuronal population and its developmental stage.

Chronic stress is one of the most important causes of depression and animals exposed to chronic GC administration exhibit increased anxiety and depressive behaviors (Gourley et al., 2008a,b). A variety of studies have implicated impaired BDNF/TrkB signaling and Erk1/2/MAPK pathway in the pathogenesis and treatment of depression (Bjorkholm and Monteggia, 2016; Castren and Kojima, 2017; Wang and Mao, 2019; Chen et al., 2020; Castren and Monteggia, 2021). For instance, the MAPK/CREB pathway is downregulated in brain regions implicated in major depression to chronic stressors, such as the prefrontal cortex and the hippocampus (Qi et al., 2008).

Moreover, BDNF signaling through TrkB is required for the behavioral effects of antidepressant drugs (Saarelainen et al., 2003; Bjorkholm and Monteggia, 2016). In fact, several antidepressant drugs normalize the BDNF-MAPK-CREB pathway in the hippocampus and induce plastic changes that ameliorate depressive symptoms (Schmidt and Duman, 2010; Bjorkholm and Monteggia, 2016; Casarotto et al., 2021). Thus, the BDNF-MAPK-CREB cascade represents a current target for developing pharmacotherapies for depression (Wang and Mao, 2019). Here, we showed that in hippocampal neurons DEX induces the expression of Sprouty4, which specifically abrogates the Ras/MAPK signaling pathway in response to BDNF. Thus, our findings suggest that Sprouty4 might be a potential target to develop antidepressant treatments. In addition, the relationship among glucocorticoids, BDNF function and negative regulators of the Erk1/2 pathway, such as Sprouty4, represents a promising avenue for understanding the contribution of neurotrophin-mediated TrkB signaling in mood disorders.

## Data availability statement

The raw data supporting the conclusions of this article will be made available by the authors, without undue reservation.

## Ethics statement

The animal study was reviewed and approved by Institutional Animal Care and Ethics Committee of the School of Medicine (CICUAL-UBA), ethical permit number: 619/2021.

## Author contributions

FFer, FFed, FL, and GP contributed to the study conception and experimental design and commented the different versions of the manuscript. Experiments, data collection and analysis were performed by FFer and FFed. The manuscript was written by GP. All authors contributed to the article and approved the submitted version.

## Funding

This work was supported by the Argentine Agency for Promotion of Science and Technology (ANPCyT; PICT2017-4513, PICT2019-1472, and PICT2017-4597). GP and FL were supported by an Independent Research Career Position from the Argentine Medical Research Council (CONICET). FFer was supported by a fellowship from ANPCyT. FFed was supported by a fellowship from CONICET.

## Conflict of interest

The authors declare that the research was conducted in the absence of any commercial or financial relationships that could be construed as a potential conflict of interest.

## Publisher’s note

All claims expressed in this article are solely those of the authors and do not necessarily represent those of their affiliated organizations, or those of the publisher, the editors and the reviewers. Any product that may be evaluated in this article, or claim that may be made by its manufacturer, is not guaranteed or endorsed by the publisher.
