# Supplementary material for: Sprouty4 at the crossroads of Trk neurotrophin receptor signaling suppression by glucocorticoids
Source: Front Mol Neurosci. 2023 Feb 2;16:1090824. doi: 10.3389/fnmol.2023.1090824 (PMC9932978; doi:10.3389/fnmol.2023.1090824)
Supplement: Supplementary file 1 [file Data_Sheet_1.PDF]

Supplementary Figures

Figure S1

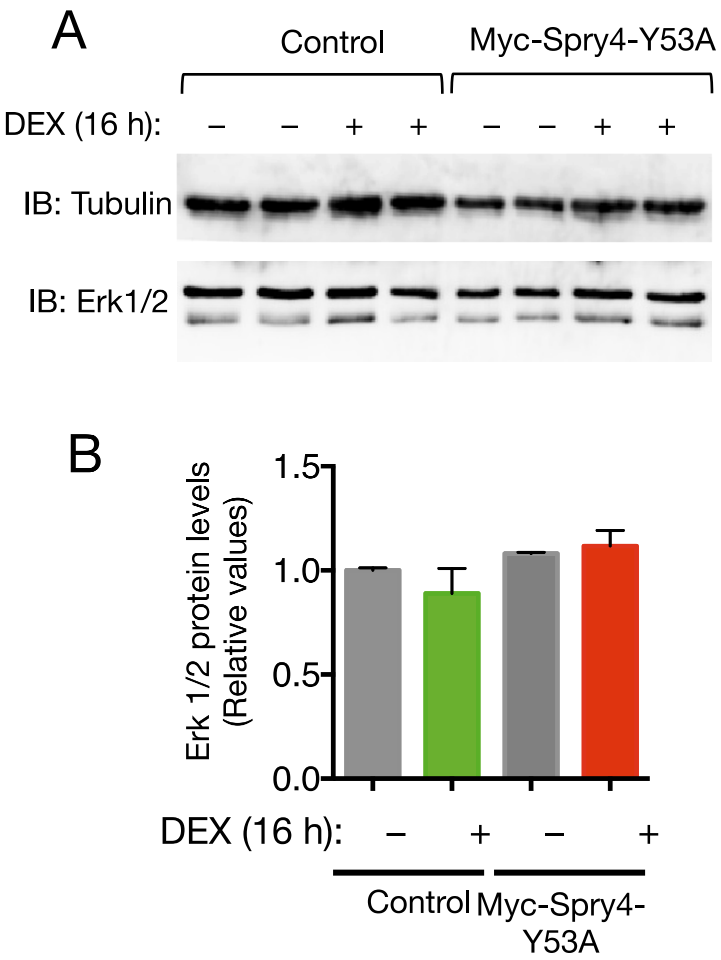

Figure S1: Neither overexpression of Spry4 Y53A nor treatment of PC12 cells with DEX affected Erk1/2/MAPK levels.

(A) Representative IB showing the levels of Erk1/2 in total extracts of control and PC12 cells overexpressing spry4 Y53A and treated or not with DEX (1 μM) for 16 h. IB of Tubulin is shown as loading control.

(B**)** Graph shows Erk1/2 protein levels normalized by Tubulin and expressed as relative to the control group untreated with DEX.

Figure S2

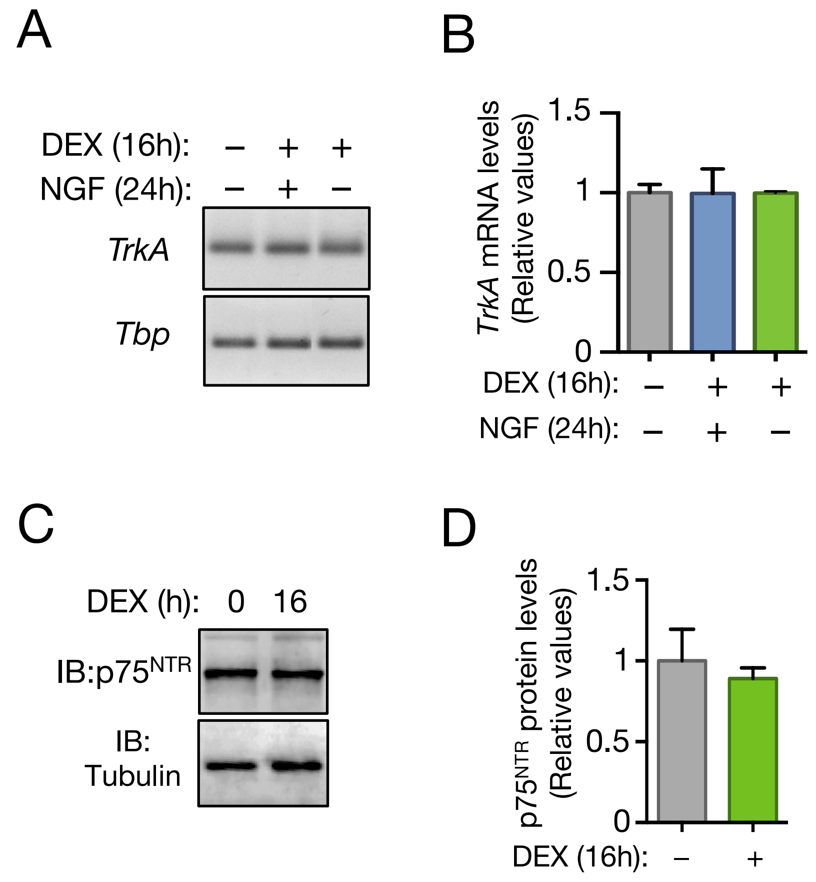

Figure S2: Treatment of PC12 cells with DEX does not affect neither *TrkA* mRNA expression nor p75^{NTR} protein levels.

(A) Semiquantitative RT-PCR analysis of *TrkA* mRNA expression in PC12 cells untreated, treated with DEX or DEX plus NGF for the indicated times.

(B) Bar graph showing the levels of *TrkA* mRNA expression among the different groups. The level of *TrkA* mRNA was normalized to the expression of the housekeeping gene *Tbp*. Values indicate fold of change of *TrkA* mRNA relative to the control untreated group.

(C) Representative IBs showing the levels of p75^{NTR} in total extracts of PC12 cells treated or not with DEX for 16 h. Tubulin is shown as loading control.

(D) Graph shows relative levels of p75^{NTR} in PC12 cell extracts treated or not with DEX for 16 h.
