# Supplementary material for: Sprouty4 at the crossroads of Trk neurotrophin receptor signaling suppression by glucocorticoids
Source: Front Mol Neurosci. 2023 Feb 2;16:1090824. doi: 10.3389/fnmol.2023.1090824 (PMC9932978; doi:10.3389/fnmol.2023.1090824)

RT-PCRs related to Figure1A

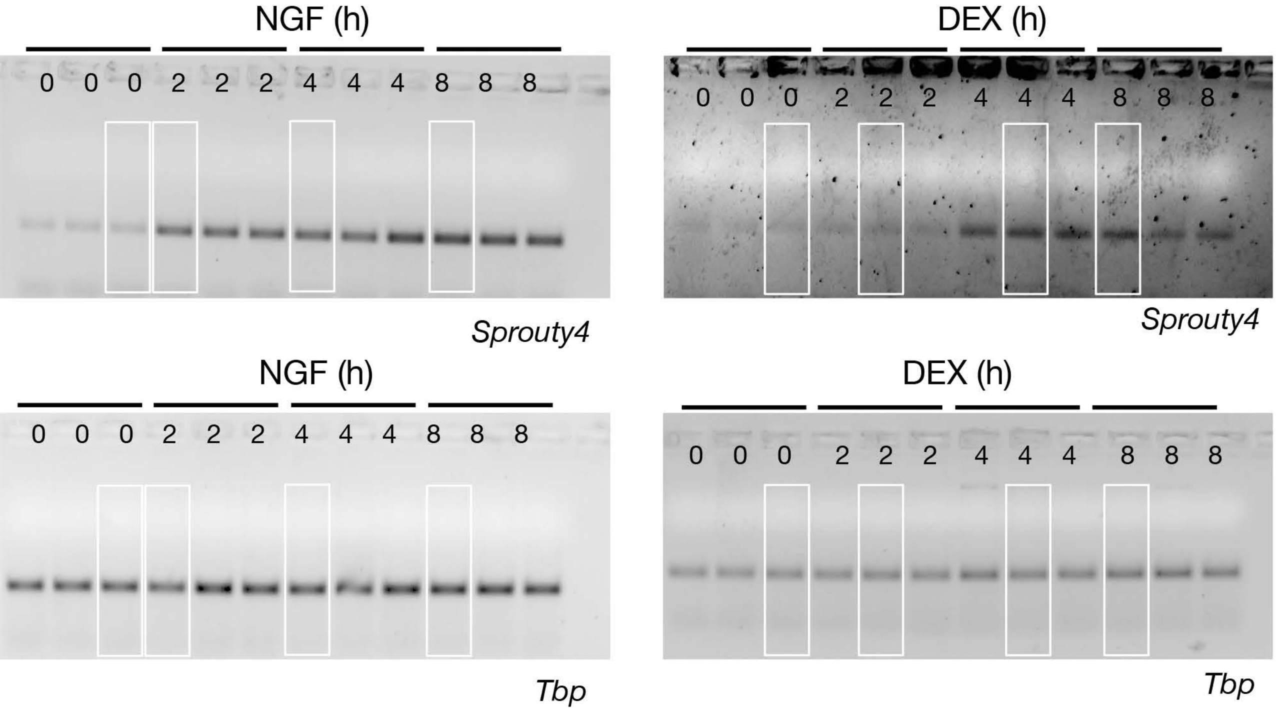

RT-PCRs related to Figure1C

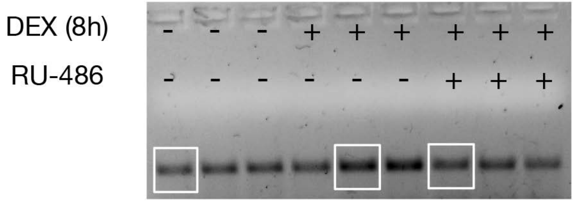

Sprouty4

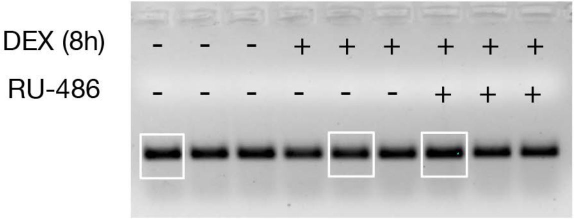

Tbp

RT-PCRs related to Figure4A

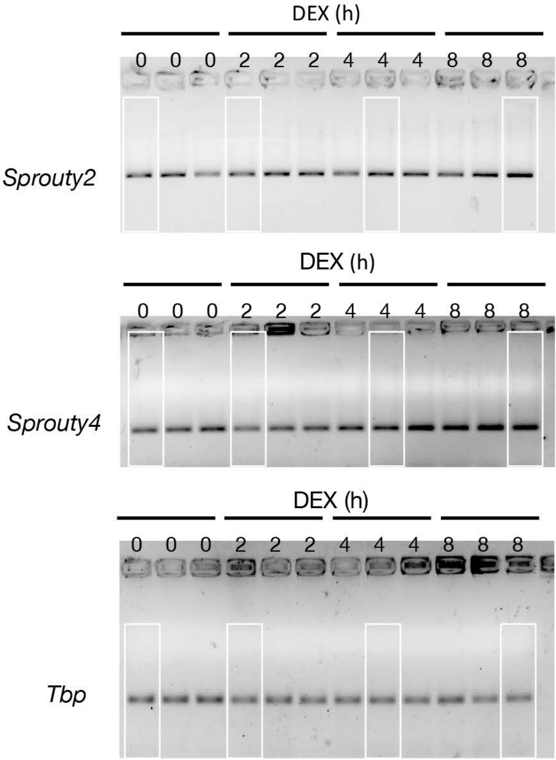

Immunoblots related to Figure 2E (oe Myc-Sprouty4 y53)

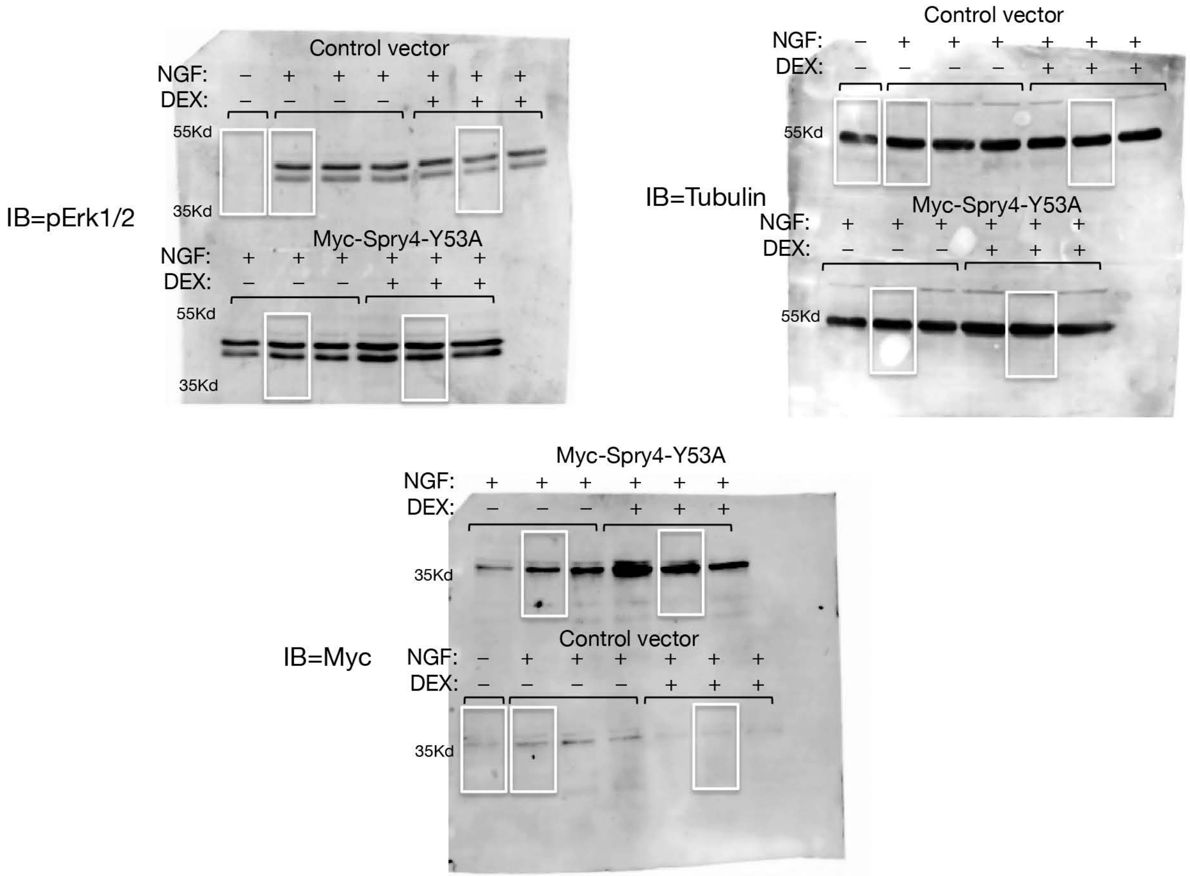

Supplement: Supplementary file 2 [file Data_Sheet_2.PDF]
